# Application of three-dimensional technology in video-assisted thoracoscopic surgery sublobectomy

**DOI:** 10.3389/fonc.2024.1280075

**Published:** 2024-03-08

**Authors:** Xinyu Zhang, Di Yang, Linqian Li, Jianing Wang, Si Liang, Peng Li, Zhe Han, Xiaodong Wang, Ke Zhang

**Affiliations:** ^1^ Clinical Medical College of Hebei University, Affiliated Hospital of Hebei University, Baoding, China; ^2^ Thoracic Surgery Department, Affiliated Hospital of Hebei University Cardiothoracic Surgical Department, Affiliated Hospital of Hebei University, Baoding, China; ^3^ Surgical Department, Affiliated Hospital of Hebei University, Baoding, China; ^4^ Basic Research Key Laboratory of General Surgery for Digital Medicine, Baoding, China; ^5^ Institute of Life Science and Green Development, Hebei University, Baoding, China; ^6^ 3D Image and 3D Printing Center, Affiliated Hospital of Hebei University, Baoding, China; ^7^ Imaging Department of Hebei University Affiliated Hospital, Baoding, China

**Keywords:** 3D reconstruction, 3D printing, sublobectomy, VATS, early lung cancer

## Abstract

**Background:**

Due to the widespread use of imaging techniques, the detection rate of early-stage lung cancer has increased. Video-assisted thoracoscopic surgery (VATS) sublobectomy has emerged as a prominent alternative to lobectomy, offering advantages like reduced resection range, better preservation of lung function, and enhanced postoperative quality of life. However, sublobectomy is more intricate than lobectomy, necessitating a higher level of surgical proficiency and anatomical understanding.

**Methods:**

Three electronic databases were searched to capture relevant studies from January 2016 to March 2023, which related to the application of three-dimensional(3D) technology in VATS sublobectomy.

**Results:**

Currently, clinical departments such as orthopedics, hepatobiliary surgery, and urology have started using 3D technology. This technology is expected to be widely used in thoracic surgery in future. Now 3D technology assists in preoperative planning, intraoperative navigation and doctor-patient communication.

**Conclusion:**

3D technologies, instrumental in locating pulmonary nodules and identifying variations in target lung segmental vessels and bronchi, play pivotal roles in VATS sublobectomy, especially in preoperative planning, intraoperative navigation, and doctor-patient communication. The limitations of 3D technology in clinical application are analyzed, and the future direction of existing 3D technology development is prospected.

## Introduction

Low-dose spiral CT is widely utilized, leading to an increased detection of ground-glass nodules (GGNs) at an early stage. In the past, anatomical lung lobectomy with mediastinal lymph node dissection has been the most commonly used gold standard treatment for early-stage non-small-cell lung cancer (NSCLC). However, lung cancer predominantly affects the elderly, with the majority of cases occurring in individuals over 60 (1). These lung cancer patients generally have poor physical conditions. Although lobectomy removes the tumor, it also removes a large amount of healthy lung tissue, which greatly affects the patient’s postoperative lung function and quality of life. Is there a more suitable and better surgical method to treat early NSCLC? Several scholars have undertaken comprehensive studies to address this question.

A series reports of the Japan Clinical Oncology Group (JCOG) 0802/0804/1211 and the latest multicenter non-inferiority verification study, such as CALGB140503 show that sublobectomy is safe and effective, which can preserve more lung function, improve the prognosis of patients with higher quality of life and longer survival time (2–5). While anatomical lobectomy remains the standard surgical procedure, sublobectomy is an optional approach for CT1N0M0 NSCLC patients who meet specific criteria (6). Although sublobectomy is a more suitable surgical option for some early lung cancer patients, it is more complex for doctors than lobectomy. It requires doctors to accurately locate tumors and identify lung segments, bronchi and pulmonary vessels that need to be removed during surgery. The requirements for doctors’ mastery of anatomy, ability to read CT images, and surgical experience are stricter. Consequently, many surgeons explore auxiliary methods to simplify the demands of VATS sublobectomy.

Initially, 3D reconstruction technology was employed in fields such as orthopedics, oral surgery, and hepatobiliary surgery. Its applications in thoracic surgery have been steadily increasing. This technology assists sublobectomy by importing patient DICOM data, like CT angiography (CTA), into 3D reconstruction software. Taking the Mimics software (Materialise, Belgium) as an example (7), first generate the three-dimensional visualization(3DV) model of the main bronchus, then add the structure of small bronchi and segment tissues such as pulmonary vasculature and lymph nodes into different masks by setting appropriate thresholds. Unnecessary tissue masks are then removed, gaps filled, modifications made, and the model is smoothed out. It’s then compared with original CT images to ensure accuracy. The final 3DV model, which includes bronchi, pulmonary vasculature, tumors, and lymph nodes, offers interactivity, allowing for enlargement, reduction, rotation, and translation. When a key observation structure needs to be highlighted, other surrounding tissues can be hidden to prevent the learner’s vision from being disturbed by irrelevant structures. Through this technology with good interactivity, the patient’s lung tumor and surrounding vessels can be more fully displayed on a two-dimensional screen before or during surgery to provide great help for the operation.

**Figure 1:**
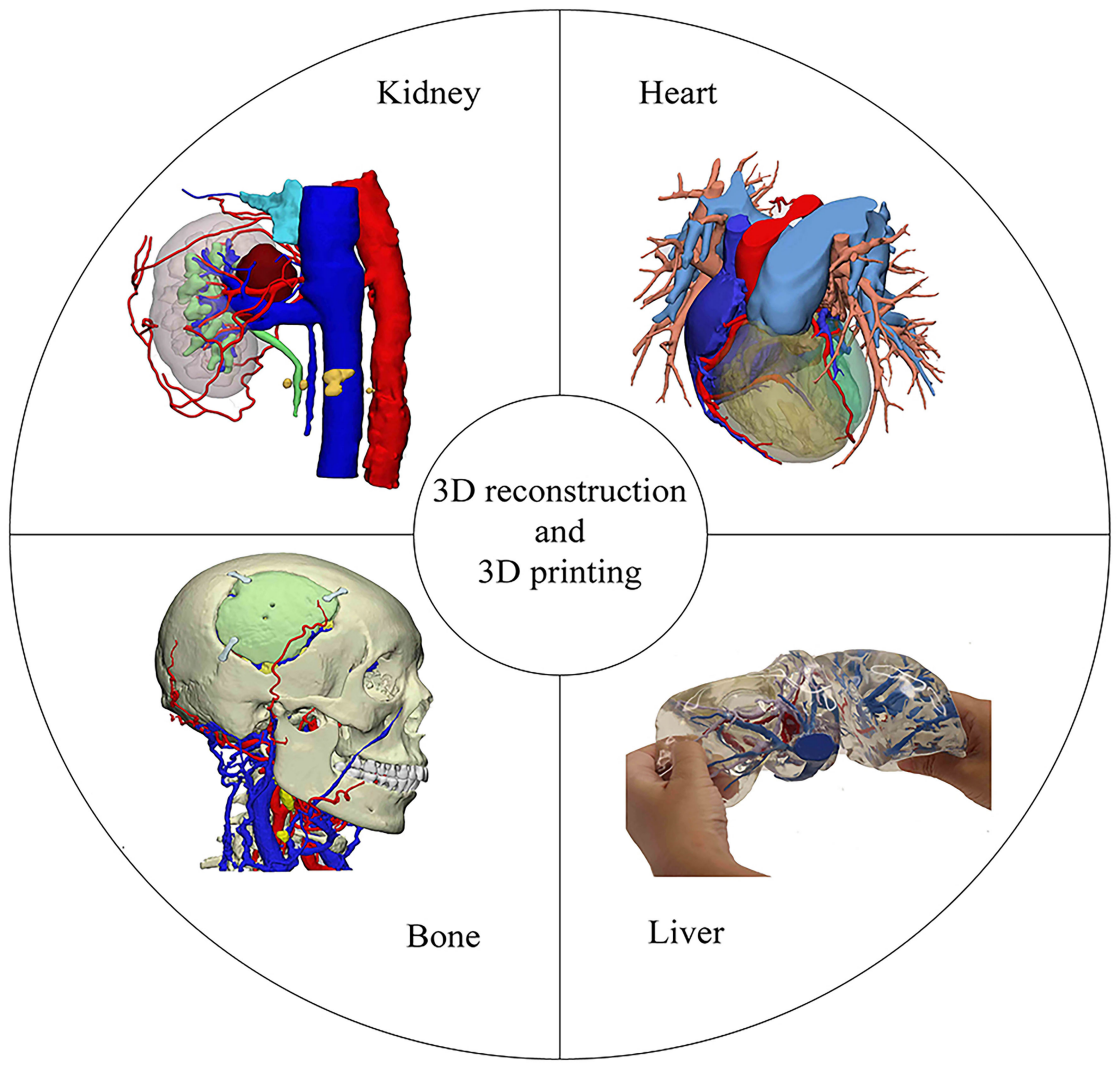
The application of 3D technology in our hospital.

While 3D reconstruction technology offers numerous advantages, it lacks the tactile feedback and immersive experience provided by physical models. Hence, with the increasing precision demands of surgeries, 3D printing technology, capable of producing tangible models, has been introduced. In recent years, 3D printing has found extensive applications in the medical domain, especially in surgical simulation, preoperative planning, and the creation of surgical assistive tools (8). Clinical departments, including orthopedics, hepatobiliary surgery, and urology, have adopted 3D printing, and its potential applications in thoracic surgery are promising (**Figure 1**).

## Materials and methods

We conducted a literature search on PubMed, Elsevier, and SpringerLink for publications from January 2016 to March 2023, focusing on the application of 3D technology in VATS sublobectomy. The search terms included: 3DV, 3D reconstruction, 3D printing, sublobectomy, lung nodule localization, lung nodule diagnosis, VATS, preoperative planning, intraoperative navigation, and doctor-patient communication. Exclusion criteria encompassed duplicate studies and those unrelated to 3D technology and VATS. The inclusion criteria targeted original articles pertinent to 3D technology and VATS. After screening titles for relevance and reviewing abstracts, studies that aligned with the research objectives and met the inclusion criteria were incorporated into our database.

## Results

**Figure 2:**
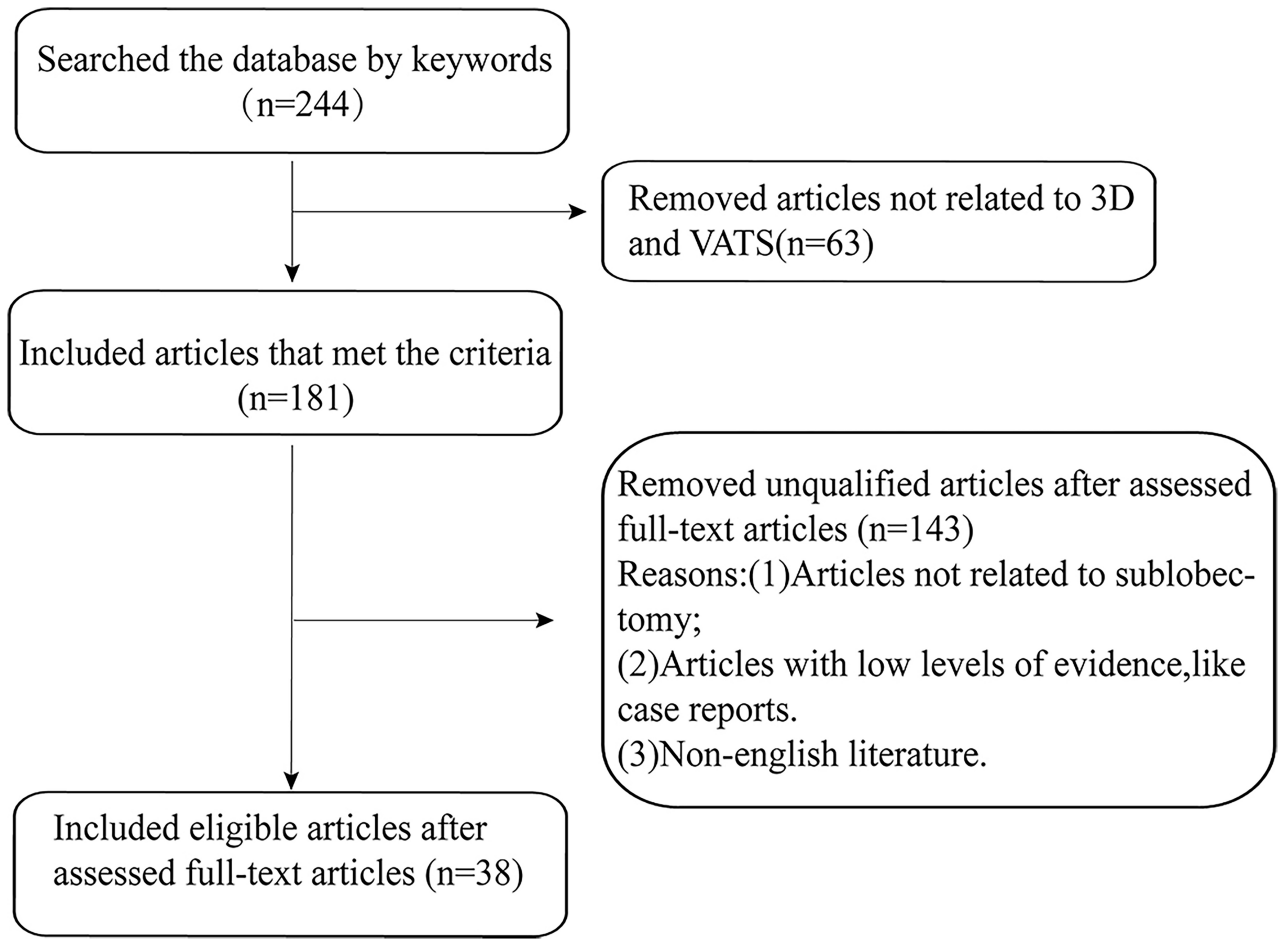
The flow diagram of articles identified and selected for inclusion in this review.

The keyword-driven database search and subsequent screening by inclusion criteria yielded an initial 244 relevant studies. Of these, 38 articles were ultimately selected for review (**Figure 2**). These studies indicated that 3D technology primarily finds application in three facets of sublobectomy: the preoperative stage, the intraoperative stage, and doctor-patient communication (**Table 1**).

**Table 1:** Application of 3D technology in sublobectomy.

Documents	Keywords	Evaluation
2022, E H, et al. (9), 2017, Zhang L, et al. (10), 2020, Fu R, et al. (11), 2019, Zhang L, et al. (12)	navigational template; preoperative nodule localization	Feasible, safe, accurate, and fast
2019, Kitano K, et al. (13), 2018, Sato M, et al. (14).	VALMAP; preoperative nodule localization	For deep pulmonary nodules, it is a minimally invasive, safe, and reliable manner.
2022, Tang H, et al. (15)	navigation template; intraoperative nodule localization	feasible
2021, Fu R, et al. (16)	navigation template; intraoperative nodule localization; MR technology	For small and deep nodules, it is useful, accurate and safe.
2021, Zhao L, et al. (17)	dial positioning method; intraoperative nodule localization	This method is accurate, safe, fast, without radiation exposure
2019, Sun W, et al. (18)	navigation template; intraoperative nodule localization; multiple pulmonary nodules.	Applicable, safe, and uncomplex
2021, Ji Y, et al. (19), 2022, Li K, et al. (20).	3D solid model; multiple pulmonary nodules	Accurate, effective and feasible.
2021, Wu X, et al. (21), 2022, Lin KH, et al. (22), 2021, Wu YJ, et al. (23)	3DV models; preoperative planning; Intraoperative navigation	It is safe and accurate for lung nodules deep or adjacent lung segment borders.
2019, Wu WB, et al. (24)	combined sub-segmentectomy; 3D reconstruction	effective
2020, Chen Y, et al. (25)	VATS segmentectomy; 3D printing; 3D reconstruction.	3D printing technology is superior to 3D reconstruction technology.
2020, Qiu B, et al. (26)	APL; 3D reconstruction; 3D printing	3D printing models are more suitable for complex segmentectomy.

### Application of 3D technology in the preoperative stage of sublobectomy

**Figure 3:**
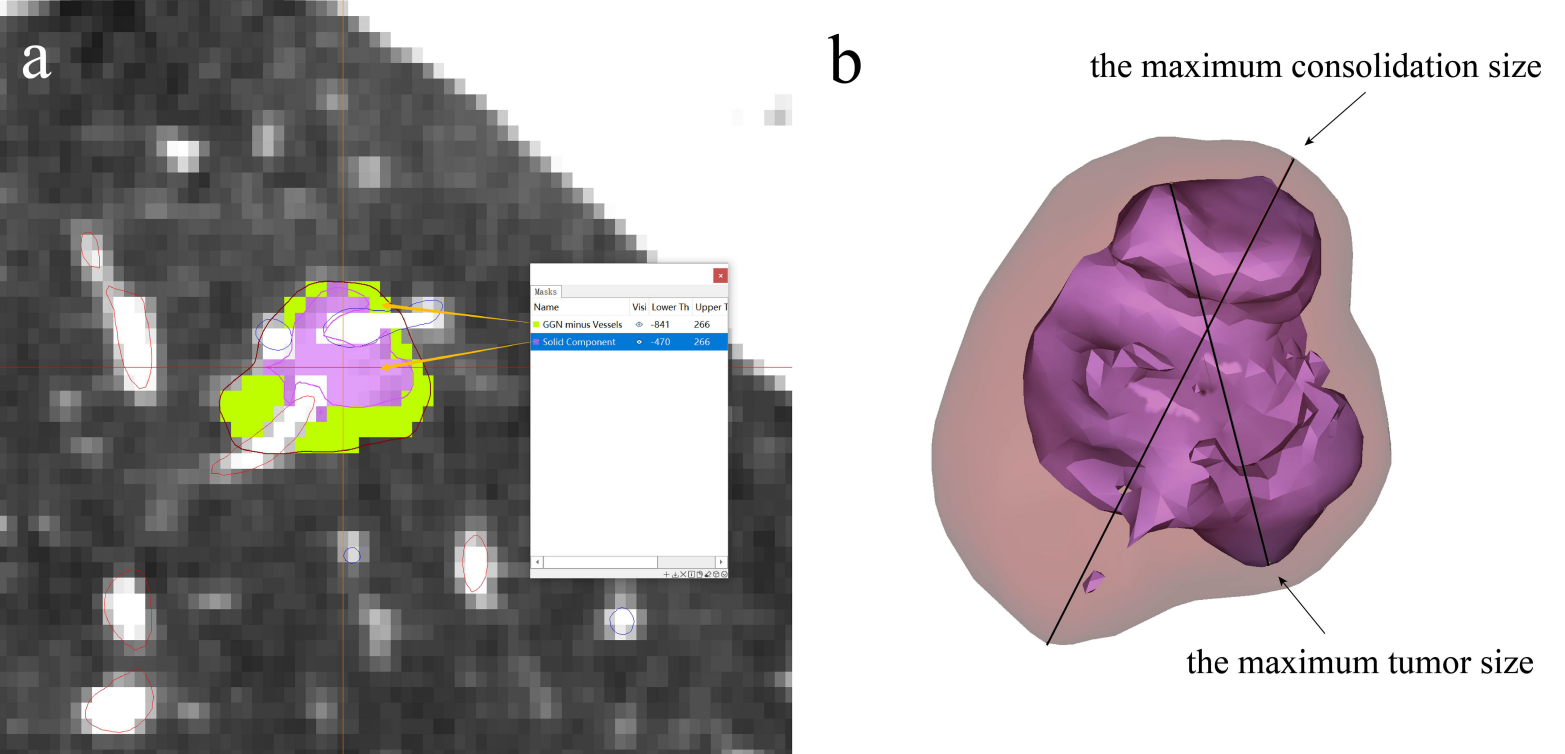
3D technology in lung nodule imaging diagnosis. **(A)** Nodule is divided into solid and ground glass component according to CT value, **(B)** CTR value can be obtained by calculating the maximum consolidation size to the maximum tumor size.

Prior to sublobectomy, accurate diagnosis of the target lung nodule is essential. Traditional clinical methods, such as fiberoptic bronchoscopy and lung puncture biopsy, are frequently employed to discern the benign or malignant nature of nodules. 3D reconstruction technology, a subset of 3D technology, facilitates the multi-angle, multi-directional 3D morphological reconstruction of lung nodules. Leveraging the 3D characteristics of nodules, along with related imaging metrics like nodule diameter, vascular bundle sign, and lobular sign, allows for a more precise assessment of nodule nature. This provides thoracic surgeons with enhanced diagnostic accuracy, aiding in the formulation of patient treatment plans (27). Moreover, 3D technology can notably enhance the success rate of preoperative lung nodule localization and planning. Lung nodules are categorized into solid and subsolid nodules. The latter is further classified into pure ground glass nodules (pGGNs) and mixed ground glass nodules (mGGNs) (28). pGGNs lack solid components and typically exhibit lower average CT values. In contrast, mGGNs comprise both solid and ground glass components, presenting with varied imaging manifestations and higher CT values. A study by Son et al., has found that increased density (75th percentile CT attenuation value on a histogram ≥-470 Hounsfield units), and entropy (a measure of heterogeneity by texture irregularity) predicted invasive adenocarcinoma (29). Therefore, we generally believe that the CT value of the solid component is higher than -470. The consolidation-to-tumor ratio (CTR) represents the ratio of the maximum consolidation size to the maximum tumor size (30). Numerous studies have affirmed the utility of CTR as a reference metric for distinguishing benign from malignant early-stage lung cancers (31). For example, JCOG 0201 has shown that the ground-glass opacity predominantly associated with excellent prognoses, and JCOG 0802 has defined radiologically non-invasive lung cancer as having a maximum tumor diameter of 2 cm with a consolidation-to-tumor ratio of 0.5 or less (2, 32). The IASLC Lung Cancer Staging Project also has found that there is a general correlation between solid patterns on CT scans and invasive patterns histologically (33). Meanwhile, according to a meta-analysis from 2023, its results suggested that higher CTR was associated with worse prognosis in NSCLC patients(the cut-off value was usually 0.5 or 0.75), and CTR can be used to predict the prognosis of NSCLC patients and guide the preoperative decision-making of patients with NSCLC (30, 34, 35). 3D measurements have identified a significant association between elevated CTR values and the invasiveness of lung adenocarcinoma (36). For T1N0M0 lung adenocarcinoma, a higher CTR value often suggests increased pathological invasiveness (37). 3D reconstruction technology not only visualizes lung nodules but also delineates their volume and CT values. The first step is to first create a tumor mask, and then remove the pulmonary arteriovenous mask so that only GGN components are left in the mask. Second, create a global mask with a threshold greater than -470.In the third step, the global mask greater than -470 is combined with the GGN mask after the removal of blood vessels to do the Boolean operation (intersection) to obtain the solid component mask. Then the GGN mask and the solid component mask are calculated as objects surrounded by triangular surfaces, and then the maximum diameter length of the GGN and the solid component is measured according to the definition calculated by CTR (**Figure 3**). This underscores the pivotal role of 3D reconstruction technology in the diagnostic differentiation of lung nodules (38).

While lung biopsy offers the highest diagnostic accuracy, it suffers from a notable limitation: a relatively high false-negative rate (39). Misdiagnoses can occur if the biopsy needle fails to intersect malignant tumor cells. 3D technology, leveraging its spatial positioning capabilities, can mitigate this limitation. Notable applications encompass 3D printing navigation template-guided lung puncture biopsy and 3D imaging-assisted bronchoscopy (9, 40), both of which demonstrate superior accuracy and safety compared to CT. Once the nature of the nodules is ascertained via biopsy, preoperative localization is typically undertaken for patients fitting surgical criteria. Currently, CT-guided Hookwire localization remains the predominant clinical method (41), but it poses risks of iatrogenic injuries and potential severe complications like pneumothorax, hemothorax, and air embolism (42). This method also demands significant technical expertise from the practitioner. Another prevalent approach is CT-guided percutaneous puncture injection using dyes. Indocyanine green, the most effective dye to date, boasts a high localization success rate and commendable safety. However, precise dosage control is crucial; excessive amounts can lead to fluorescence dispersion in the pleural cavity, while insufficient quantities might result in failed localization (43). Furthermore, these methods might not accurately pinpoint the lung segment housing the nodule or delineate the surgical safety margin for nodules.

**Figure 4:**
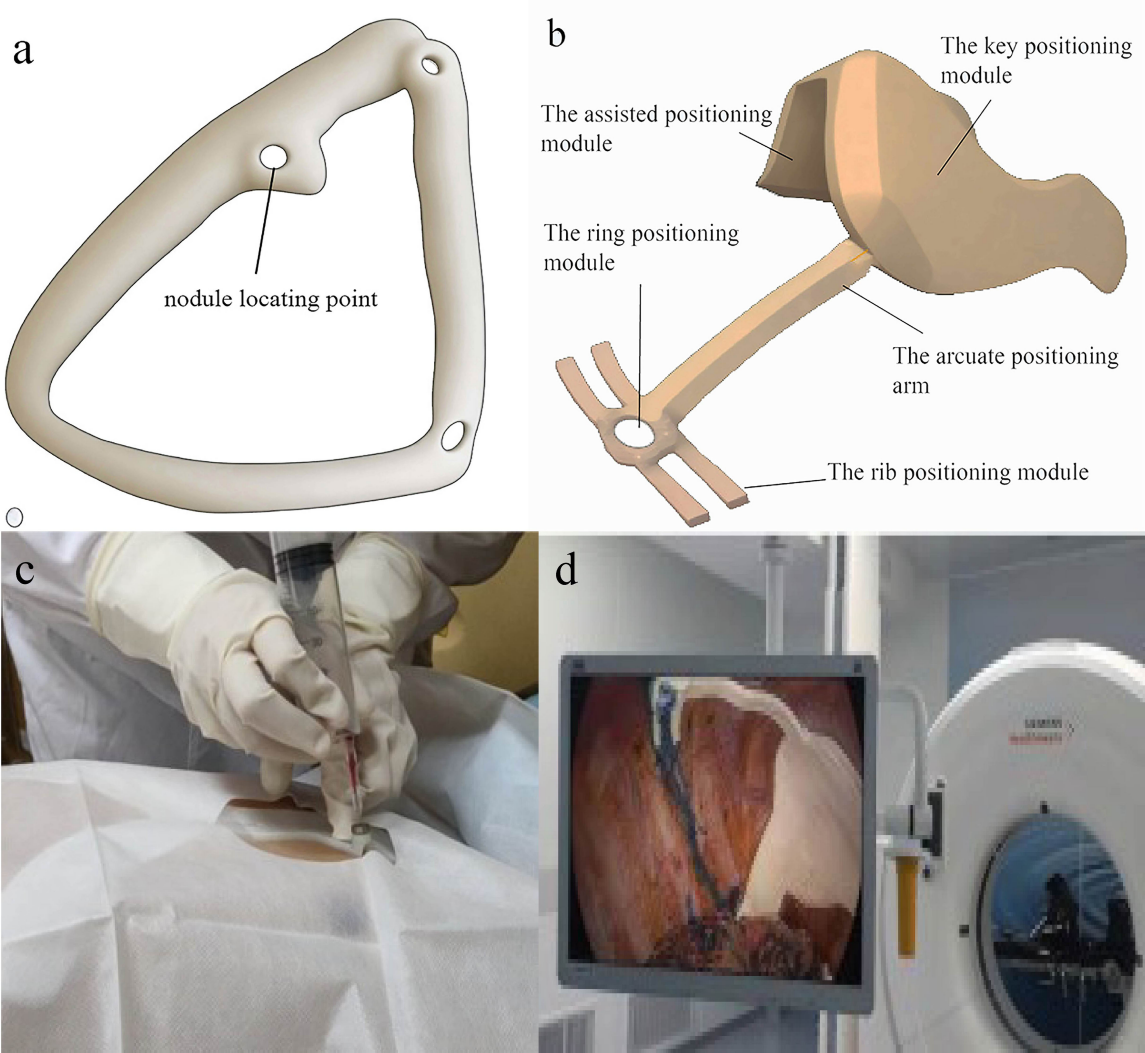
3D navigational template in preoperative and intraoperative lung nodule localization. **(A)** 3D preoperative navigational template. Surgeon puncture at the nodule locating point ; **(B)** 3D intraoperative navigational template. The key positioning module will be placed on the top of the pleural cavity and match with some anatomic landmark. according to the assisted positioning module and rib positioning module, the nodule will be located by the ring positioning module. Then doctor stain the corresponding location. **(C)** 3D preoperative navigational template in surgery. It is quoted from reference 22. **(D)** 3D intraoperative navigational template made by TPU in surgery. It is quoted from reference 37.

In comparison to traditional CT-guided puncture localization, which has inherent limitations such as radiation exposure and accuracy influenced by nodule depth, 3D technology offers significant advantages in assisting lung nodule localization (44). A prevalent method involves reconstructing a digital pulmonary model, followed by designing and printing a 3D physical navigation template for preoperative localization (**Figures 4A, C**) (10). This navigation template typically aligns with anatomical markers, clearly indicating the direction, position, angle, and depth of the puncture point. Physicians can then perform punctures swiftly and simply along the designated tract (11). The success rate for template-guided puncture stands at 89%, markedly surpassing that of CT-guided puncture (6.3%). This method also considerably reduces positioning time and radiation exposure (P < 0.001) (12). Beyond the 3D navigation template, 3D technology offers a myriad of applications in aiding preoperative localization of pulmonary nodules. One notable technique is virtual-assisted lung mapping (VALMAP), rooted in 3D reconstruction technology, which proves invaluable for resecting multiple deep-seated pulmonary nodules. Preoperatively, a 3DV model of virtual bronchoscopy guides the procedure. Markings are made on the visceral pleura using dye injections under bronchoscopy, followed by 3D reconstruction to verify the accuracy of the marking range (13). However, mastering this method poses challenges, and potential severe complications like hypertension and hypoxemia can arise. Additionally, if the nodule’s position is too deep, the likelihood of localization failure escalates (14). 3D technology also facilitates preoperative planning and simulation through virtual reality (VR) systems (45). Integrating conventional imaging modes with VR systems significantly enhances preoperative planning, bolstering the safety and precision of anatomical resection.

Research indicates that 3D technology equips physicians with a more comprehensive understanding of surgical intricacies preoperatively compared to conventional methods, simultaneously minimizing unnecessary preoperative trauma.

### Application of 3D technology in the intraoperative stage of sublobectomy

While anatomical studies highlight the coexistence of bronchus and corresponding pulmonary artery, with the pulmonary vein traversing between segmental planes, variations in bronchus and pulmonary vessels are frequently observed across patients (46). Solely relying on standard pulmonary anatomy, without considering vascular variations evident in preoperative CT images, can lead to surgical complications such as erroneous ligation or intraoperative vascular injury. Moreover, certain nodules, due to their diminutive size, depth, and inaccessibility, pose challenges for intraoperative localization and resection (47). Traditional methods often fall short in addressing these challenges. In contrast to conventional 2D data sources like anatomical atlases and CT scans, 3D models vividly depict the lungs’ three-dimensional internal and external structures, offering surgeons a more comprehensive, intuitive, and tangible grasp of the surgical region. To enhance nodule localization accuracy, delineate a safer resection boundary, mitigate the risk of inadvertent vascular injuries, and elevate surgical precision and safety, 3D technology has been integrated into the intraoperative navigation of VATS sublobectomy (48).

**Figure 5:**
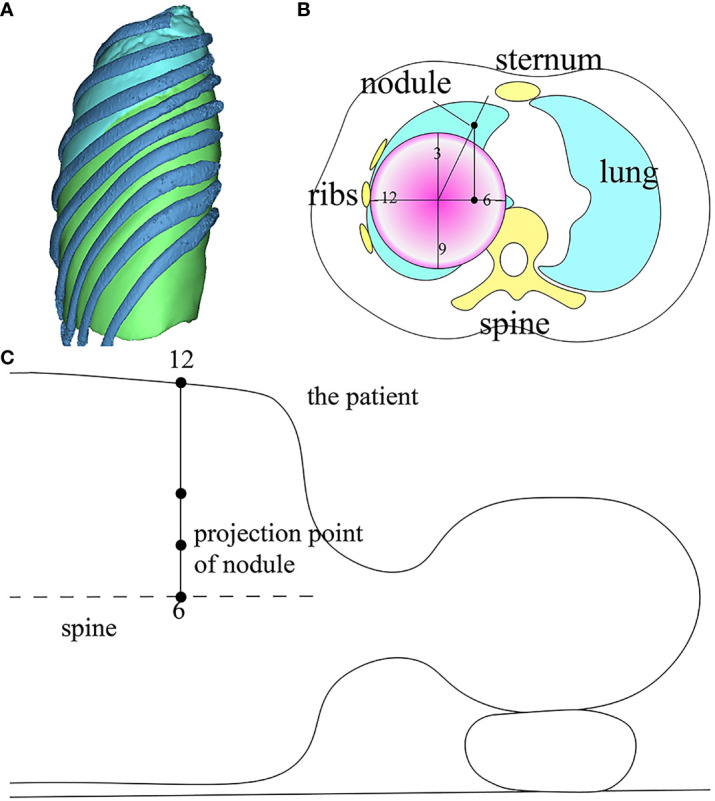
three-dimensional reconstruction combined with dial positioning in intraoperative lung nodule localization. **(A)** Reconstruct lung and ribs, and record the precise positional relationship between the nodule and the nearest rib; **(B)** Locate the plane of pulmonary nodule on horizontal CT, and draw a circular dial on the CT of affected lung. Record the orientation of the nodule. **(C)** Draw the horizontal lines of CT cross-resection and mark the projection point of nodule across the patient’s body, then puncture and mark on the lung surface.

Certain nodules, due to their depth, present heightened risks associated with preoperative puncture. Additionally, some patients decline invasive preoperative examinations or localizations. Consequently, a significant number of nodules undergo resection without preoperative puncture. In VATS sublobectomy, the restricted access of the operation hole complicates direct nodule confirmation. To address this, 3D physical navigation templates crafted from thermoplastic polyurethanes (TPU) are employed. These templates can be introduced into the pleural cavity without preoperative puncture, facilitating intraoperative nodule localization and resection (15), thereby circumventing puncture-related complications and streamlining the localization process (**Figures 4B, D**). Intraoperative localization offers additional benefits: it eliminates the need to transfer patients from the radiology department to the operating room, conserves time, and spares patients from preoperative anxiety due to general anesthesia (11). 3D printed navigation templates can further integrate with mixed reality (MR) technology. Preoperatively, MR glasses allow surgeons to view a 3D-reconstructed virtual thoracic holographic projection. Using 3D printed navigation templates for tactile feedback, the puncture’s point, angle, and depth can be ascertained in the operating room. This combined approach is viable even for impalpable lung nodules, eliminating patient transfers between CT and operating rooms (16). Other innovative applications of 3D technology for intraoperative lung nodule localization have emerged. For instance, Zhao et al. introduced a dial positioning method (**Figure 5**) (17). This real-time intraoperative technique not only mitigates complications like hemothorax and pneumothorax but also operates independently of CT assistance, ensuring zero radiation exposure.

**Figure 6:**
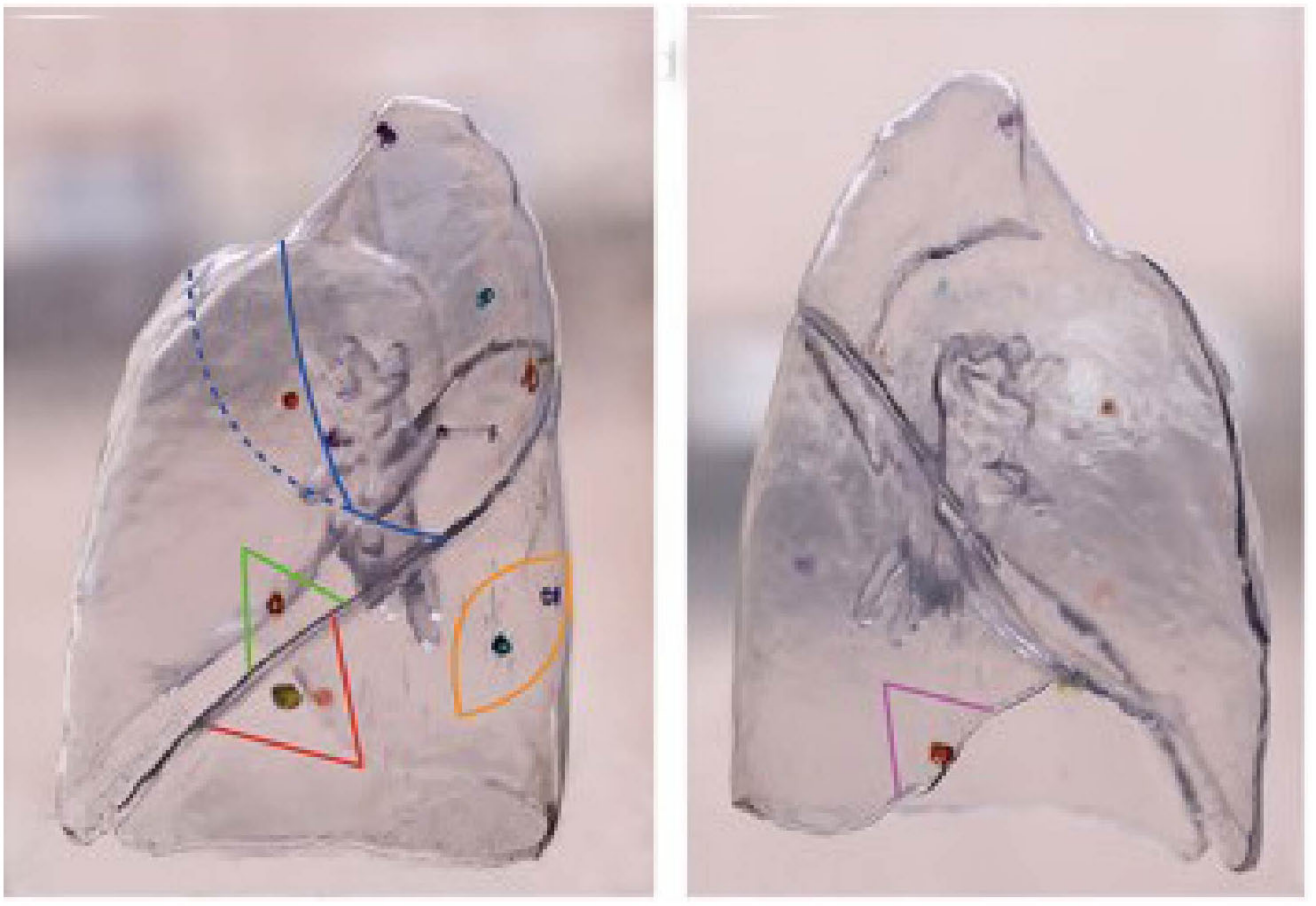
3D printing technology in locating lung nodules. It is quoted from reference 43.

Current methods for localizing multiple lung nodules, such as employing markers like Hookwire for percutaneous puncture localization, necessitate multiple scans, proving to be intricate and time-intensive. Moreover, the recurrent insertion of localization devices can elevate the risk of pneumothorax, complicating the achievement of swift, safe, and effective localization. While electromagnetic navigation bronchoscopy (ENB) ensures a commendable success rate and safety for localization, it is predominantly utilized for solitary nodules and demands significant technical expertise from the operator (49). Conversely, 3D technology-assisted localization obviates the need for supplementary examination equipment and intricate operational procedures. After identifying the pulmonary vessels in the target region through preoperative 3D reconstruction and determining the pulmonary segment via the inflation-deflation method, surgeons can proceed directly to resection. 3D physical navigation templates can guide intraoperative localization of multiple lung nodules without relying on CT equipment (18). Additionally, direct reconstruction and 3D printing of physical models of multiple lung nodules can aid in preoperative planning and intraoperative navigation. The nodule positions depicted by the 3D printed model offer greater precision and clarity than pathological reports (19). Furthermore, 3D printed models can distinctly represent minor lesions that remain ambiguous in terms of benignity or malignancy, assisting physicians in assessing the surgical resection scope. Surgeons can opt to resect these minor nodules concurrently if they don’t interfere with the primary surgical plan, potentially excising up to 12 nodules in one procedure (20) (**Figure 6**). If any of these nodules prove malignant, the patient can avoid a secondary surgery post-lung cancer recurrence, thereby preventing significant physical and economic repercussions.

Before undertaking a sublobectomy, it’s imperative not only to pinpoint the location of lung nodules but also to comprehend the intricate anatomical structures of the neighboring sublobar units, encompassing arteries, veins, and bronchi (50). This knowledge facilitates the demarcation of sublobar unit boundaries, assessment of the spatial relationship between the nodule and the sublobar unit, and determination of the specific sublobar region for resection. Unlike 2D imaging modalities such as CT and MRI, 3D technology transforms these 2D images into comprehensive 3D representations of vascular and bronchial trees, effectively delineating vessel branching patterns and accentuating anatomical variations in both vessels and bronchi (51). Subsequent analysis of intersegmental veins within these sublobar anatomical units allows for precise boundary identification. Moreover, this technology vividly illustrates the spatial relationship between the nodule and adjacent vessels, aiding in the accurate judgment and resection of target sublobar tissues while ensuring surgical margin safety.

For segmentectomy procedures, utilizing 3D reconstruction software, such as 3D Slicer and Mimics, for preoperative reconstruction and simulation offers advantages over traditional CT images. This method facilitates the creation of surgical plans and provides intraoperative navigation, proving especially beneficial for the precise excision of deep nodules or those situated along the borders of adjacent segments. Through the 3DV model, surgeons can ascertain the spatial relationship between nodules and segment borders, thereby determining the safe resection margin distance. This enhances surgical precision and safety, ensures adequate surgical margins (21), diminishes the recurrence rate of lung cancer (22), and preserves ample normal lung tissue. The 3DV model also provides a detailed visualization of the blood vessels and bronchi within the target lung segment, aiding surgeons in discerning the spatial relationship between the nodule, bronchus, and pulmonary vasculature. This clarity ensures accurate segment resection and minimizes the risk of intersegmental vein injury (23). Notably, for variant blood vessels and bronchi, surgical safety has seen marked improvement. Beyond static 3DV models, Tokuno J et al. introduced a semi-automatic simulation system for dynamic 3D images, capturing the intraoperative lung deformation (52).

**Figure 7:**
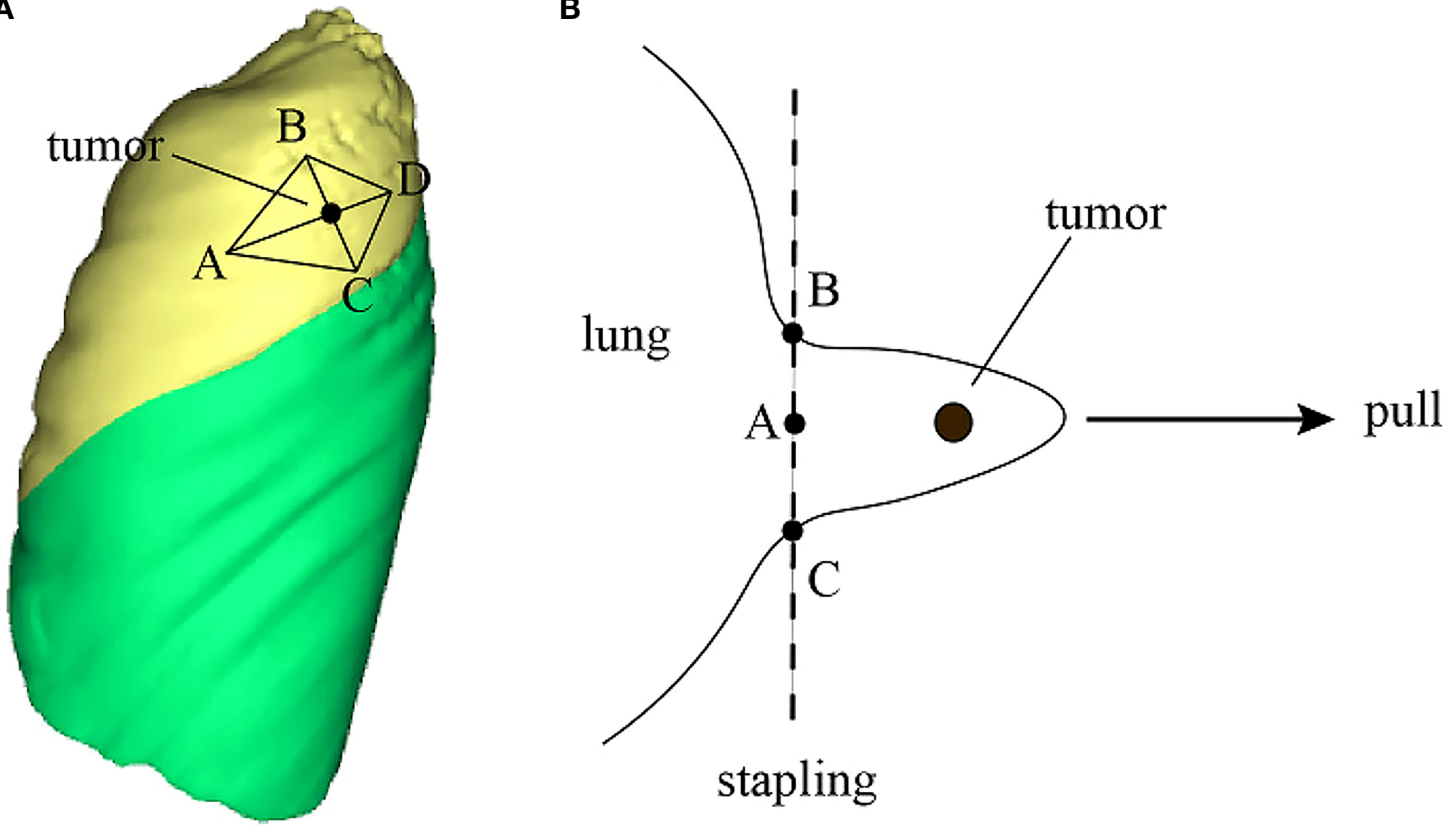
3D technology to improve surgical operation safety and quality. **(A)** Reconstruct the 3DV model and locate pulmonary nodule and draw a rhomboidal cut line and four marker points on the 3DV model; **(B)** According to the 3DV model, CT image and anatomical landmarks, mark correct points on the pleura, then resect the lung nodule along the cut line by a stapler.

In wedge resection, 3D reconstruction technology offers significant intraoperative assistance. Using the 3DV model, physicians can identify the clipping points based on the nodule’s safe margins and adjacent blood vessels. Subsequently, the anticipated surgical incision line and resection plane for the wedge resection are delineated. The appropriate points are then marked on the lung using a marker pen, guiding the wedge resection (**Figure 7**) (53). This method streamlines preoperative planning, facilitates the estimation of lung tissue thickness for stapler use, and aids in selecting the appropriate stapler types or assessing the feasibility of a wedge resection (53).

Beyond segmentectomy and wedge resection, sublobectomy encompasses the more intricate combined sub-segmentectomy (51). This procedure is primarily tailored for lung nodules situated between lung segments. The surgical target area combines two adjacent lung subsegments with the nodule and neighboring intersegmental veins (54). As the surgical focus narrows from lung segments to subsegments, the procedure’s complexity increases, necessitating meticulous preoperative planning. This ensures surgeons have a comprehensive understanding of the boundaries of the subsegments adjacent to the nodule and can determine the subsegments requiring resection. Compared to segmentectomy and wedge resection, the successful execution of combined sub-segmentectomy is more dependent on 3D technology for preoperative planning and intraoperative navigation (24).

Relative to 3DV models, 3D printed models excel in pinpointing nodules and delineating intricate vascular structures. These models not only enhance the success rate of nodule localization and resection but also surpass 3D reconstruction technology in terms of reducing surgical conversion rates (0% vs 10.5%), operation duration (2.07 ± 0.24h vs 2.55 ± 0.41h), and intraoperative blood loss (43.25 ± 13.63 mL vs 96.68 ± 32.82 mL) (P<0.05) (25). This superiority stems from the fact that 3D printed models offer surgeons a tangible 3D perspective during preoperative planning, facilitating the identification of nodules and the intricate network of blood vessels and bronchi, without the need for mental visualization. Qiu Bin et al. highlighted the pronounced benefits of both 3D reconstruction and 3D printed models in discerning vascular variations during anatomical partial-lobectomy (APL) (26). They further underscored the spatial and distance accuracy of 3D printed models, emphasizing their suitability for complex segmentectomy and the notable reduction in operation time compared to 3D reconstructed models.

Collectively, these studies underscore that 3D technologies, encompassing both 3D reconstruction and 3D printing, render VATS sublobotomy more rapid, safe, and efficient than conventional techniques, with 3D printing technology holding an edge over 3D reconstruction.

### Application of 3D technology in doctor-patient communication

Lung cancer predominantly affects the elderly, a demographic often unfamiliar with medical intricacies. Given the specialized nature of medical professionals, a cognitive gap exists between doctors and patients, complicating effective communication. To secure informed consent for VATS sublobectomy, it’s imperative that patients and their families grasp the tumor’s location and size, the surgical approach, and potential postoperative complications. 3D technology, particularly 3D printing, can produce tangible models of a patient’s lungs, offering a tangible medium for doctor-patient dialogue (55). The tangible nature of these models significantly enhances communication. For instance, a survey of early-stage lung cancer patients, where some were presented with 3D printed models during the informed consent process, indicated that these models enriched the patients’ comprehension of their ailment (56). Another survey of surgeons utilizing 3D printed models as surgical aids revealed that 88% felt the models improved communication with patients and their families (26). This preference might stem from the tangible, stereoscopic nature of 3D printed models, which might resonate more with patients and their kin compared to 3DV models. Such studies suggest that 3D technology in doctor-patient communication can bridge the understanding gap, fostering a more collaborative doctor-patient dynamic. This approach might also mitigate potential doctor-patient conflicts and bolster the overall doctor-patient relationship. Based on our hospital’s medical 3D printing center’s experience, 3D printed models, when utilized in surgical planning, intraoperative navigation, and doctor-patient communication, yield superior results compared to 3D reconstructed digital models.

## Conclusions

As advanced technology inevitably supersedes outdated methods, sublobectomy is poised to gain broader acceptance. Similarly, the closely related 3D technology will play an increasingly important role in thoracic surgery department.

Although 3D technology currently serves as an excellent auxiliary tool for VATS sublobectomy, it still has some limitations. First, the precision and comprehensiveness of models generated by 3D reconstruction hinge on the creator’s expertise in interpreting CT images and their grasp of anatomy. Moreover, there is currently no structured training program specifically tailored for medical professionals in 3D technology. The thoracic surgery field urgently needs to establish quality control standards for 3D reconstruction and 3D printing and needs to stipulate to what level of refinement the reconstruction or printing can meet clinical application. According to the experience of the medical 3D printing center in our hospital, after reconstruction, the contours need to be verified through medical software certified by the medical software management department. If the reconstructed tissue is one level higher than the part involved in the surgery, the model is considered to meet the minimum requirements of the surgery. Second, the cost of 3D printing might be prohibitive for many patients. However, if one opts to 3D print only the physical navigation templates rather than the entire lung model, the cost is considerably reduced, averaging between $75-90 (9).The high cost of 3D printing technology impacts not just patients but also hospitals, encompassing expenses related to the purchase and maintenance of 3D printers, material costs for the models, and other factors that hinder its broad adoption. 3D reconstruction and printing are time-consuming processes; designing and printing a basic 3D physical navigation template can take between three to five hours (10). It takes longer to 3D print a complete model. When it comes to lung models, producing a qualified model can span 3-4 days.

In the realm of early lung cancer detection, 3D technology is anticipated to evolve towards cost and time efficiency. With ongoing advancements in 3D reconstruction and printing, challenges like high material costs and extended printing durations are expected to be addressed. Emerging artificial intelligence technologies are streamlining the adoption of 3D reconstruction techniques and enhancing the efficiency of 3DV model creation. This trend suggests a potential evolution in 3D technology towards greater intelligence and automation, which could significantly streamline the 3D reconstruction process and reduce manual input. According to the experience of our hospital, AI has better effects in the thoracic surgery field compared with other systems. This might be attributed to the inherent good contrast between pulmonary vascular tissue and the surrounding lung tissue. Thus, with the ongoing advancements in medical imaging technology and surgical techniques, we anticipate that 3D reconstruction and 3D printing-assisted VATS sublobectomy will have a promising future in the realm of early lung cancer treatment.

## Data availability statement

Publicly available datasets were analyzed in this study. This data can be found here: http://pubmed.ncbi.nlm.nih.gov.

## Author contributions

XZ: Writing – original draft. LL: Writing – review & editing. DY: Data curation, Writing – review & editing. ZH: Supervision, Writing – review & editing. XW: Supervision, Writing – review & editing. JW: Writing – review & editing. PL: Supervision, Writing – review & editing. SL: Writing – review & editing. KZ: Funding acquisition, Supervision, Writing – review & editing

## Supplementary material

Supplementary Video 1 A presentation video of 3D reconstruction
